# Double Asymptotic Expansion of Three-Center Electron Repulsion Integrals 2nd Derivatives

**DOI:** 10.1021/acs.jctc.6c00784

**Published:** 2026-07-08

**Authors:** F. A. Olvera-Rubalcava, G. Geudtner, A. M. Köster, P. Calaminici

**Affiliations:** Chemistry Department, CINVESTAV, Av. Instituto Politécnico Nacional, 2508, Col. San Pedro Zacatenco, Del. Gustavo A. Madero, C.P. 07360 Mexico City, Mexico

## Abstract

The calculation of the skeleton Hessian in the framework of auxiliary density functional theory (ADFT) requires the computation of the second derivatives of three-center electron repulsion integrals (ERIs). Because the evaluations of ERIs and their derivatives involve the calculations of the nonanalytic Boys functions, this calculation step can become a computational bottleneck for large molecular systems. To avoid this bottleneck the double asymptotic expansion of three-center ERIs was proposed. In this work, we extend this expansion to analytic second derivatives of these ERIs. To this end, the working equations for the double asymptotic expansion of three-center ERI second derivatives are derived. They are implemented in the analytic second energy derivative calculation in the ADFT branch of deMon2k. To assess their computational performance skeleton Hessian matrix benchmark calculations of linear alkane chains, carbon fullerenes, DNA fragments and hydrogen saturated mobil-5 type (ZSM-5) zeolite fragments with up to 43,000 basis functions are presented.

## Graphical abstract

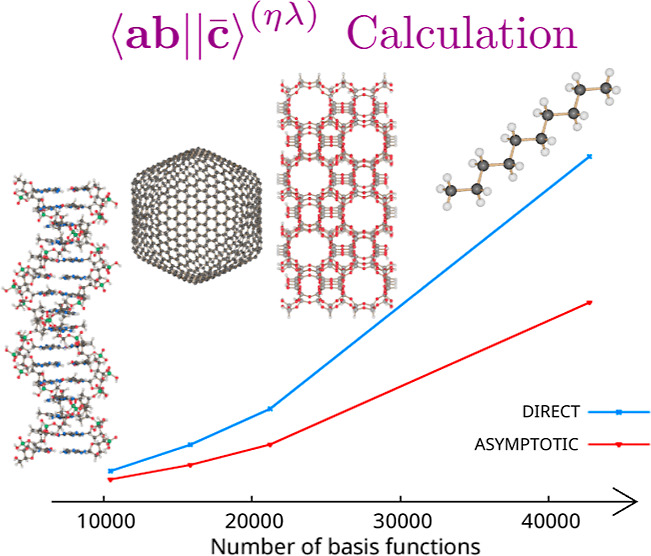

## 1 Introduction

With the rise of low-order scaling Hartree–Fock^1,2^ and Kohn–Sham^3,4^ methods the efficient calculation of three-center electron repulsion integrals (ERIs) gains increasing importance. To this end, the structure of the underlying auxiliary basis set (ABS) must be taken into account. Although, it is often assumed that the ABS structure follows the orbital basis set (OBS), this is neither necessary nor beneficial for the calculation of the three-center ERIS. In fact, the advantage of primitive Hermite–Gaussian auxiliary basis functions with shared exponents for the recursive calculation of the three-center ERIs has been explored already many years ago.^5–7^ The accuracy and reliability of automatically generated ABSs with these structures have been recently demonstrated.^8^ Despite these efforts, the brute force three-center ERI calculation with recurrence relations becomes a bottleneck in low-order scaling Hartree–Fock and Kohn–Sham calculations of large molecular systems with many hundreds or thousands of atoms.^9^ Fortunately, for large systems with weakly overlapping orbital densities simplifications can be introduced. When two electron densities do not exhibit significant overlap, that is, their respective centers are far apart, they can be considered to lie in each others asymptotic region. In this situation, an asymptotic expansion can be used to precisely calculate the interaction energy of the two electron densities. To this end, the expansion of the two-electron Coulomb operator into a multipole series is commonly used.^10^ For the efficient computation of long-range Coulomb interactions the fast multipole method (FMM) from Greengard and Rokhlin^11^ is advocated. In the FMM the system is divided into boxes that define regions of near and far neighbors. The adaption of the FMM to electronic structure calculations for molecular as well as for periodic systems is well-documented in the literature.^12–19^ An attractive alternative to the FMM is the double asymptotic ERI expansion^20^ that avoids the explicit spatial division of the molecule. For primitive Hermite Gaussian ABS the double asymptotic three-center ERI expansion yields particularly simple expressions that show subquadratic scaling with respect to the number of basis functions in the systems. So far the double asymptotic three-center ERI expansion is implemented in deMon2k^21^ for energy and gradient calculations. With the extension of auxiliary density functional theory (ADFT)^22^ to the calculation of analytic second energy derivatives with respect to nuclear displacements λ and η,^23,24^ which we will denote by *E* ^(λη)^, the second derivatives of the three-center ERIs are needed. These second energy derivatives are important for structure optimization and characterization. Their calculation augments the basis set angularity by two units and, therefore, can become a bottleneck in frequency analyses of larger molecular systems.

To circumvent this bottleneck, we present in this work the double asymptotic expansion for three-center ERI second derivatives. To validate and benchmark these equations, we implemented them in the ADFT branch of deMon2k for the analytic calculation of the skeleton Hessian matrix. This work is organized as follows. In the next section the working equations for the double asymptotic expansion of three-center ERI second derivatives are derived. To put this work in perspective, we briefly review in Subsection [Sec sec2.1] the analytic second ADFT energy derivatives and in Subsection [Sec sec2.2] the double asymptotic expansion of three-center ERIs employing primitive Hermite Gaussian auxiliary functions. The working equations for the double asymptotic expansion of the three-center ERI second derivatives are derived in Subsection [Sec sec2.3] the analytic second ADFT energy derivatives and in Subsection [Sec sec2.2] the double asymptotic expansion of three-center ERIs . The following section gives the computational details for the skeleton Hessian matrix benchmark calculations of linear alkane chains, carbon fullerenes, DNA fragments and hydrogen saturated mobil-5 type (ZSM-5) zeolite fragments. The benchmark results are discussed in Section 4 and conclusions are summarized in the last section.

## 2 Theoretical Background

### 2.1 Analytic Second ADFT Energy Derivatives

For the sake of simplicity, we restrict our discussion to closed-shell systems where occupied spatial molecular orbitals (MOs) are each doubly occupied. Employing the linear combination of the Gaussian-type orbitals (LCGTO) approximation, the ADFT energy expression can be written as^22,25^

$$E=\underset{a,b}{\sum }P_{ab}H_{ab}+\underset{a,b}{\sum }\underset{\overset{®}{c}}{\sum }P_{ab}\left\langle ab∥\mathrm{c̅}\right\rangle x_{\mathrm{c̅}}-\frac{1}{2}\underset{\mathrm{c̅},\mathrm{d̅}}{\sum }x_{\mathrm{c̅}}x_{\mathrm{d̅}}\left\langle \mathrm{c̅}∥\mathrm{d̅}\right\rangle +E_{xc}[\mathrm{\rho ̃}].$$ 1

In eq 1, *P*_ab_ denotes the density matrix elements. The core matrix elements, *H*_ab_, collect the contributions of the kinetic energy of electrons and nuclear attraction energy. The second and third terms of the right side of eq 1 represent the Coulomb repulsion energy between the electrons, arising from the variational fitting of this energy.^26–29^ The last term of eq 1 is the exchange–correlation energy evaluated with the variationally fitted auxiliary density. The variational fitting of the Coulomb energy is based on the minimization of the second-order error functional

$$\varepsilon_{2}^{H}=\frac{1}{2}\int \int \frac{\left[\rho \left({\mathrm{r⃗}}_{1}\right)-\mathrm{\rho ̃}\left({\mathrm{r⃗}}_{1}\right)\right]\left[\rho \left({\mathrm{r⃗}}_{2}\right)-\mathrm{\rho ̃}\left({\mathrm{r⃗}}_{2}\right)\right]}{\left|{\mathrm{r⃗}}_{1}-{\mathrm{r⃗}}_{2}\right|}d{\mathrm{r⃗}}_{1}d{\mathrm{r⃗}}_{2}.$$ 2

In eq 2, the electron density, ρ(r⃗) , is given by

3 $$\rho \left(\mathrm{r⃗}\right)=2\sum_{i}^{occ}\sum_{a,b}c_{ai}c_{bi}a\left(\mathrm{r⃗}\right)b\left(\mathrm{r⃗}\right)=\sum_{a,b}P_{ab}a\left(\mathrm{r⃗}\right)b\left(\mathrm{r⃗}\right),$$

with

$$P_{ab}=2\sum_{i}^{occ}c_{ai}c_{bi}.$$

Here, *c*_*ai*_ and *c*_*bi*_ denote MO coefficients. The atomic orbitals (AOs), **a** (r⃗) and **b** (r⃗) , are expressed as Cartesian contracted GTOs, centered at atom $\mathrm{A⃗}=\left(A_{x},A_{y},A_{z}\right)$ , according to

$$a\left(\mathrm{r⃗}\right)=\overset{K_{a}}{\underset{k=1}{\sum }}d_{k}a_{k}\left(\mathrm{r⃗}\right).$$ 5

The (unnormalized) primitive GTOs are given by

$$a_{k}={\left(x-A_{x}\right)}^{a_{x}}{\left(y-A_{y}\right)}^{a_{y}}{\left(z-A_{z}\right)}^{a_{z}}e^{-\zeta_{k}{\left(\mathrm{r⃗}-\mathrm{A⃗}\right)}^{2}}.$$ 6

The triad of non-negative integer numbers, (*a*_*x*_, *a*_*y*_, *a*_*z*_), is the angular momentum index of the AO, *K*_*a*_ is the contraction degree, *d*_*k*_ are the contraction coefficients and ζ_*k*_ are the primitive GTO exponents.

The auxiliary density, ρ̃(r⃗) , in eq 2 is expanded as given in ref:^6^

$$\mathrm{\rho ̃}\left(\mathrm{r⃗}\right)=\underset{\overset{®}{c}}{\sum }x_{\mathrm{c̅}}\mathrm{c̅}\left(\mathrm{r⃗}\right).$$ 7

In eq 7 c̅(r⃗) denotes atom-centered primitive Hermite Gaussian auxiliary functions at atom C⃗ = (*C*_*x*_, *C*_*y*_, *C*_*z*_) with angular momentum index $\left({\mathrm{c̅}}_{x},{\mathrm{c̅}}_{y},{\mathrm{c̅}}_{z}\right)$ , and exponent $\zeta_{\mathrm{c̅}}$ given by

$$\mathrm{c̅}\left(\mathrm{r⃗}\right)={\left(\frac{\partial }{\partial C_{x}}\right)}^{{\mathrm{c̅}}_{x}}{\left(\frac{\partial }{\partial C_{y}}\right)}^{{\mathrm{c̅}}_{y}}{\left(\frac{\partial }{\partial C_{z}}\right)}^{{\mathrm{c̅}}_{z}}e^{-\zeta_{\mathrm{c̅}}{\left(\mathrm{r⃗}-\mathrm{C⃗}\right)}^{2}}.$$ 8

The corresponding Coulomb fitting coefficients are denoted by $x_{\mathrm{c̅}}$ . The fitting coefficients are obtained by solving the linear equation system arising from the minimization of the error functional in eq 2

9 Gx=J

The elements of the Coulomb matrix, G, and Coulomb vector, **J**, are

10 $$G_{\mathrm{c̅}\mathrm{d̅}}=\left\langle \mathrm{c̅}∥\mathrm{d̅}\right\rangle ,,J_{\mathrm{c̅}}=\sum_{a,b}P_{ab}\left\langle ab∥\mathrm{c̅}\right\rangle$$

The double bar symbol, ∥, in the here used two- and three-center ERI short-hand notation represents the two-electron Coulomb operator, $1/\left|{\mathrm{r⃗}}_{1}-{\mathrm{r⃗}}_{2}\right|$ . It also separates the functions of electron 1 in the bra from those of electron 2 in the ket.

The contracted ERIs, which are denoted by angular brackets, can be calculated from the corresponding uncontracted ERIs, denoted by squared brackets, according to

$$\left\langle ab∥\mathrm{c̅}\right\rangle =\overset{K_{a}}{\underset{k}{\sum }}\overset{K_{b}}{\underset{l}{\sum }}d_{k}d_{l}\left[a_{k}b_{l}∥\mathrm{c̅}\right].$$ 11

Finally, the last term of eq 1 is the exchange–correlation energy, evaluated with the auxiliary density.

The analytic second derivatives of the ADFT energy expression given in eq 1 with respect to the atomic coordinates λ and η, taking into account the perturbation dependency of the OBS and ABS, are given by^23,24^

$$\begin{array}{ll} E^{\left(\lambda \eta \right)} & =\underset{a,b}{\sum }P_{ab}^{\left(\eta \right)}\left(H_{ab}^{\left(\lambda \right)}+\underset{\overset{®}{c}}{\sum }{\left\langle ab∥\mathrm{c̅}\right\rangle }^{\left(\lambda \right)}\left(x_{\mathrm{c̅}}+z_{\mathrm{c̅}}\right)\right)+\\ & \underset{a,b}{\sum }P_{ab}\left(H_{ab}^{\left(\lambda \eta \right)}+\underset{\overset{®}{c}}{\sum }{\left\langle ab∥\mathrm{c̅}\right\rangle }^{\left(\lambda \eta \right)}\left(x_{\mathrm{c̅}}+z_{\mathrm{c̅}}\right)\right)+\\ & \underset{a,b}{\sum }\underset{\overset{®}{c}}{\sum }P_{ab}{\left\langle ab∥\mathrm{c̅}\right\rangle }^{\left(\lambda \right)}\left(x_{\mathrm{c̅}}^{\left(\eta \right)}+z_{\mathrm{c̅}}^{\left(\eta \right)}\right)+\underset{\overset{®}{c}}{\sum }x_{\mathrm{c̅}}^{\left(\eta \right)}\left\langle {\mathrm{c̅}}^{\left(\lambda \right)}|v_{xc}\left[\mathrm{\rho ̃}\right]\right\rangle +\\ & \underset{\overset{®}{c}}{\sum }x_{\mathrm{c̅}}\left\langle {\mathrm{c̅}}^{\left(\lambda \eta \right)}|v_{xc}\left[\mathrm{\rho ̃}\right]\right\rangle +\underset{\mathrm{c̅},\mathrm{d̅}}{\sum }x_{\mathrm{c̅}}\left\langle {\mathrm{c̅}}^{\left(\lambda \right)}\left|f_{xc}\left[\mathrm{\rho ̃}\right]\right|\mathrm{d̅}\right\rangle x_{\mathrm{d̅}}^{\left(\eta \right)}+\\ & \underset{\mathrm{c̅},\mathrm{d̅}}{\sum }x_{\mathrm{c̅}}\left\langle {\mathrm{c̅}}^{\left(\lambda \right)}\left|f_{xc}\left[\mathrm{\rho ̃}\right]\right|{\mathrm{d̅}}^{\left(\eta \right)}\right\rangle x_{\mathrm{d̅}}-\underset{a,b}{\sum }W_{ab}^{\left(\eta \right)}S_{ab}^{\left(\lambda \right)}-\underset{a,b}{\sum }W_{ab}S_{ab}^{\left(\lambda \eta \right)}-\\ & \underset{\mathrm{c̅},\mathrm{d̅}}{\sum }G_{\mathrm{c̅}\mathrm{d̅}}^{\left(\lambda \eta \right)}x_{\mathrm{d̅}}\left(\frac{1}{2}x_{\mathrm{c̅}}+z_{\mathrm{c̅}}\right)-\underset{\mathrm{c̅},\mathrm{d̅}}{\sum }G_{\mathrm{c̅}\mathrm{d̅}}^{\left(\lambda \right)}\left(x_{\mathrm{c̅}}+z_{\mathrm{c̅}}\right)x_{\mathrm{d̅}}^{\left(\eta \right)}-\underset{\mathrm{c̅},\mathrm{d̅}}{\sum }G_{\mathrm{c̅}\mathrm{d̅}}^{\left(\lambda \right)}z_{\mathrm{c̅}}^{\left(\eta \right)}x_{\mathrm{d̅}}. \end{array}$$ 12

In eq 12 superscripts in parentheses denote derivatives with respect to the atomic coordinates λ and η, e.g. $E^{\left(\lambda \eta \right)}\equiv \frac{\partial^{2}E}{\partial \lambda \partial \eta }$ .

The ADFT exchange–correlation potential, $v_{xc}\left[\mathrm{\rho ̃}\left(\mathrm{r⃗}\right)\right]$ , and kernel, $f_{xc}\left[\mathrm{\rho ̃}\left(\mathrm{r⃗}\right)\right]$ , are the first and second functional derivatives, respectively, of the exchange–correlation energy functional with respect to the auxiliary density.^30^ For the local density approximation (LDA) and generalized gradient approximation (GGA) they are given according to^30^ as

$$v_{xc}\left[\mathrm{\rho ̃}\left(\mathrm{r⃗}\right)\right]\equiv \frac{\delta E_{xc}\left[\mathrm{\rho ̃}\left(\mathrm{r⃗}\right)\right]}{\delta \mathrm{\rho ̃}\left(\mathrm{r⃗}\right)},,\frac{\delta v_{xc}\left[\mathrm{\rho ̃}\left({\mathrm{r⃗}}_{1}\right)\right]}{\delta \mathrm{\rho ̃}\left({\mathrm{r⃗}}_{2}\right)}\equiv f_{xc}\left[\mathrm{\rho ̃}\left({\mathrm{r⃗}}_{1}\right);{\mathrm{r⃗}}_{2}\right]\delta ({\mathrm{r⃗}}_{1}-{\mathrm{r⃗}}_{2}).$$ 13

The exchange–correlation fitting coefficients, $z_{\mathrm{c̅}}$ , are defined by^22^

$$z_{\mathrm{c̅}}=\underset{\overset{®}{d}}{\sum }G_{\mathrm{c̅}\mathrm{d̅}}^{-1}\left\langle \mathrm{d̅}|v_{xc}\left[\mathrm{\rho ̃}\right]\right\rangle .$$ 14

In eq 12, *S*_*ab*_ denotes overlap matrix elements and *W*_*ab*_ are the energy-weighted density matrix elements given by

$$W_{ab}=2\overset{occ}{\underset{i}{\sum }}\varepsilon_{i}c_{ai}c_{bi}.$$ 15

For the further discussion, it is convenient to sort the individual terms in eq 12 according to their dependency on perturbed matrix or vector elements, namely the perturbed density matrix, **P** ^(η)^, the perturbed energy-weighted density matrix, **W** ^(η)^, the perturbed Coulomb fitting coefficients, **x** ^(η)^, and the perturbed exchange–correlation fitting coefficients, **z** ^(η)^. Based on this sorting, the second-order ADFT energy derivative can be partitioned as^23,24^

$$E^{\left(\lambda \eta \right)}=E^{\left(\lambda \eta \right)}+E^{\left(\lambda \eta \right)}$$ 16

The first contribution, named the skeleton Hessian, $E^{\left(\lambda \eta \right)}$ , is completely independent of the perturbed matrix and vector elements. It has the form

$$\begin{array}{ll} E^{\left(\lambda \eta \right)} & =\underset{a,b}{\sum }P_{ab}\left(H_{ab}^{\left(\lambda \eta \right)}+\underset{\overset{®}{c}}{\sum }{\left\langle ab∥\mathrm{c̅}\right\rangle }^{\left(\lambda \eta \right)}\left(x_{\mathrm{c̅}}+z_{\mathrm{c̅}}\right)\right)+\underset{\overset{®}{c}}{\sum }x_{\mathrm{c̅}}\left\langle {\mathrm{c̅}}^{\left(\lambda \eta \right)}|v_{xc}\left[\mathrm{\rho ̃}\right]\right\rangle +\\ & \underset{\mathrm{c̅},\mathrm{d̅}}{\sum }x_{\mathrm{c̅}}\left\langle {\mathrm{c̅}}^{\left(\lambda \right)}\left|f_{xc}\left[\mathrm{\rho ̃}\right]\right|{\mathrm{d̅}}^{\left(\eta \right)}\right\rangle x_{\mathrm{d̅}}-\underset{a,b}{\sum }W_{ab}S_{ab}^{\left(\lambda \eta \right)}-\underset{\mathrm{c̅},\mathrm{d̅}}{\sum }G_{\mathrm{c̅}\mathrm{d̅}}^{\left(\lambda \eta \right)}x_{\mathrm{d̅}}\left(\frac{1}{2}x_{\mathrm{c̅}}+z_{\mathrm{c̅}}\right). \end{array}$$ 17

The analytic molecular integral derivatives in eq 17 can be straightforwardly calculated by integral recurrence relations extended to second derivatives. Similarly, the numerical integrals of the exchange–correlation potential and kernel are evaluated with the same adaptive grid that is used in the self-consistent field (SCF) calculation. Note that the primitive Hermite Gaussian auxiliary function derivatives are simply high-order functions of the same type. The exchange–correlation kernel is calculated either by analytic expressions for the kernel or by finite differences if only analytic exchange–correlation potential expressions are available.^31^ The density, *P*_*ab*_, and energy-weighted density, *W*_*ab*_, matrix elements are calculated from the SCF converged canonical MO coefficients and energies. The fitting coefficients, $x_{\mathrm{c̅}}$ and $z_{\mathrm{c̅}}$ , are also taken directly from the previously converged single-point energy calculation.

The other contribution to the second-order derivatives of the ADFT energy, $E^{\left(\lambda \eta \right)}$ , contains only terms that depend on the perturbed matrix or vector elements

$$\begin{array}{ll} E^{\left(\lambda \eta \right)} & =\underset{a,b}{\sum }P_{ab}^{\left(\eta \right)}\left(H_{ab}^{\left(\lambda \right)}+\underset{\overset{®}{c}}{\sum }\langle ab∥{\mathrm{c̅}\rangle }^{\left(\lambda \right)}\left(x_{\mathrm{c̅}}+z_{\mathrm{c̅}}\right)\right)+\\ & \underset{a,b}{\sum }\underset{\overset{®}{c}}{\sum }P_{ab}\left\langle ab∥{\mathrm{c̅}\rangle }^{\left(\lambda \right)}\left(x_{\mathrm{c̅}}^{\left(\eta \right)}+z_{\mathrm{c̅}}^{\left(\eta \right)}\right)+\underset{\overset{®}{c}}{\sum }x_{\mathrm{c̅}}^{\left(\eta \right)}\langle {\mathrm{c̅}}^{\left(\lambda \right)}|v_{xc}\left[\mathrm{\rho ̃}\right]\right\rangle +\\ & \underset{\mathrm{c̅},\mathrm{d̅}}{\sum }x_{\mathrm{c̅}}\langle {\mathrm{c̅}}^{\left(\lambda \right)}\left|f_{xc}\left[\mathrm{\rho ̃}\right]\right|\mathrm{d̅}\rangle x_{\mathrm{d̅}}^{\left(\eta \right)}-\underset{a,b}{\sum }W_{ab}^{\left(\eta \right)}S_{ab}^{\left(\lambda \right)}-\underset{\mathrm{c̅},\mathrm{d̅}}{\sum }G_{\mathrm{c̅}\mathrm{d̅}}^{\left(\lambda \right)}\left(x_{\mathrm{c̅}}+z_{\mathrm{c̅}}\right)x_{\mathrm{d̅}}^{\left(\eta \right)}-\\ & \underset{\mathrm{c̅},\mathrm{d̅}}{\sum }G_{\mathrm{c̅}\mathrm{d̅}}^{\left(\lambda \right)}z_{\mathrm{c̅}}^{\left(\eta \right)}x_{\mathrm{d̅}}. \end{array}$$ 18

Again, the molecular integral derivatives can be straightforwardly calculated. For the calculation of the perturbed $x_{\mathrm{c̅}}^{\left(\eta \right)}$ vector elements, auxiliary density perturbation theory (ADPT)^32,33^ is employed. Once the perturbed vector elements are at hand the calculation of the perturbed density matrix, *P*_*ab*_^(η)^, and perturbed energy-weighted density matrix, *W*_*ab*_^(η)^, elements is straightforward.^23^

As eq 17 shows, the three-center ERI second derivatives ${\left\langle ab∥\mathrm{c̅}\right\rangle }^{\left(\lambda \eta \right)}$ introduce a formal cubic scaling into the skeleton Hessian calculations. Particularly, for large systems with many atoms the calculation of these derivatives becomes the computationally dominant task due to the efficient grid point screening in the numerical integration of the ADFT kernel integrals $\left\langle {\mathrm{c̅}}^{\left(\lambda \right)}\left|f_{xc}\left[\mathrm{\rho ̃}\right]\right|{\mathrm{d̅}}^{\left(\eta \right)}\right\rangle$ . To overcome this bottleneck, we propose in this work the double asymptotic expansion of the three-center ERI second derivatives. To this end, we now briefly review the double asymptotic expansion of three-center ERIs.

### 2.2 Double Asymptotic Expansion of Three-Center ERIs

The three-center ERIs are given by

$$\left\langle ab∥\mathrm{c̅}\right\rangle =\int \int \frac{a\left({\mathrm{r⃗}}_{1}\right)b\left({\mathrm{r⃗}}_{1}\right)\mathrm{c̅}\left({\mathrm{r⃗}}_{2}\right)}{\left|{\mathrm{r⃗}}_{1}-{\mathrm{r⃗}}_{2}\right|}d{\mathrm{r⃗}}_{1}d{\mathrm{r⃗}}_{2}=\int a\left({\mathrm{r⃗}}_{1}\right)b\left({\mathrm{r⃗}}_{1}\right)\phi_{\mathrm{c̅}}\left({\mathrm{r⃗}}_{1}\right)d{\mathrm{r⃗}}_{1}.$$ 19

In eq 19, $\phi_{\mathrm{c̅}}\left({\mathrm{r⃗}}_{1}\right)$ is the electrostatic potential of the primitive Hermite Gaussian type function, defined as

$$\phi_{\mathrm{c̅}}\left({\mathrm{r⃗}}_{1}\right)=\int \frac{\mathrm{c̅}\left({\mathrm{r⃗}}_{2}\right)}{\left|{\mathrm{r⃗}}_{1}-{\mathrm{r⃗}}_{2}\right|}d{\mathrm{r⃗}}_{2}.$$ 20

As shown in,^20^ the asymptotic expansion of the above three-center electron repulsion integrals yields nuclear attraction-like integrals of the form

$$\left\langle ab∥\mathrm{c̅}\right\rangle ∼{\left(\frac{\pi }{\zeta_{\mathrm{c̅}}}\right)}^{3/2}\left\langle ab|{Â}_{C}\left(\mathrm{c̅}\right)\right\rangle .$$ 21

In eq 21, the nuclear attraction-like operator, ${Â}_{C}$ , is given as

$${Â}_{C}\left(\mathrm{c̅}\right)={\left(\frac{\partial }{\partial C_{x}}\right)}^{{\mathrm{c̅}}_{x}}{\left(\frac{\partial }{\partial C_{y}}\right)}^{{\mathrm{c̅}}_{y}}{\left(\frac{\partial }{\partial C_{z}}\right)}^{{\mathrm{c̅}}_{z}}\frac{1}{\left|\mathrm{r⃗}-\mathrm{C⃗}\right|}.$$ 22

The nuclear attraction-like integrals in eq 21 still demand the calculation of the Boys function.^34^ However, they already show a reduced scaling compared to the ERIs of eq 19 due to their one-electron nature. To avoid completely the time demanding evaluation of the Boys function a second asymptotic expansion, now for the nuclear attraction-like integrals,^35^ can be performed. This yields the double asymptotic expansion of the ERIs given by^20^

$$\left\langle ab∥\mathrm{c̅}\right\rangle ∼{\left\langle ab∥\mathrm{c̅}\right\rangle }^{A}+{\left\langle ab∥\mathrm{c̅}\right\rangle }^{B}.$$ 23

In eq 23 ${\left\langle ab∥\mathrm{c̅}\right\rangle }^{A}$ and ${\left\langle ab∥\mathrm{c̅}\right\rangle }^{B}$ are the far-field ERIs expanded at center A⃗ , for primitive GTO products $a_{k}\left(\mathrm{r⃗}\right)b_{l}\left(\mathrm{r⃗}\right)$ closer to this center, or at center B⃗ , for primitive GTO products closer to center B⃗ . The far-field ERIs are given by

$${\left\langle ab∥\mathrm{c̅}\right\rangle }^{A}={\left(\frac{\pi }{\zeta_{\mathrm{c̅}}}\right)}^{3/2}\underset{m}{\sum }M\left(m\right)T_{AC}\left(m+\mathrm{c̅}\right)\left\langle a+m|b\right\rangle$$ 24

and

$${\left\langle ab∥\mathrm{c̅}\right\rangle }^{B}={\left(\frac{\pi }{\zeta_{\mathrm{c̅}}}\right)}^{3/2}\underset{m}{\sum }M\left(m\right)T_{BC}\left(m+\mathrm{c̅}\right)\left\langle a|b+m\right\rangle$$ 25

with

26 $$M\left(m\right)=\frac{{\left(-1\right)}^{m}}{m_{x}!m_{y}!m_{z}!},,T_{AC}\left(m\right)={\left(\frac{\partial }{\partial C_{x}}\right)}^{m_{x}}{\left(\frac{\partial }{\partial C_{y}}\right)}^{m_{y}}{\left(\frac{\partial }{\partial C_{z}}\right)}^{m_{z}}\frac{1}{\left|\mathrm{A⃗}-\mathrm{C⃗}\right|}$$

$$m+\mathrm{c̅}=\left(m_{x}+{\mathrm{c̅}}_{x},m_{y}+{\mathrm{c̅}}_{y},m_{z}+{\mathrm{c̅}}_{z}\right),,a+m=\left(a_{x}+m_{x},a_{y}+m_{y},a_{z}+m_{z}\right).$$ 27

In these equations, the sum over **m** is a short-hand notation for the triple sum over *m*_*x*_, *m*_*y*_ and *m*_*z*_. These sums run according to *m* = *m*_*x*_ + *m*_*y*_ + *m*_*z*_ from *m* = 0 to *m* = 8.^20^ As these equations show, double asymptotic expanded ERIs are free of Boys function evaluations. Instead, two center overlap-type integrals are needed. Their separation from the diatomic Cartesian tensors, *T*_*AC*_ and *T*_*BC*_, further reduces the scaling. The definition of the diatomic Cartesian tensor for center A⃗ , *T*_*AC*_, is given in eq 26. *T*_*BC*_ is defined analogously for center B⃗ . For a more detailed discussion of the double asymptotic ERI expansion and a comparison with the fast multipole method we refer the interested reader to.^20^ The double asymptotic expansion can be applied according to the asymptotic potential radius, *r*_ϕ_, and the AO radius, *r*_χ_. The asymptotic potential radius is implemented as

$$r_{\phi }=\left|\mathrm{r⃗}-\mathrm{C⃗}\right|=\sqrt{\frac{-a-\min\left(\ln\;\tau ,-10\right)}{b,\zeta_{\mathrm{c̅}}^{\min}}}.$$ 28

Here, $\zeta_{\mathrm{c̅}}^{\min}$ is the smallest auxiliary basis set exponent at atom C⃗ . The constants are given as *a* = 2.038972 and *b* = 1.069227. For atomic orbitals, finite extension radii are defined according to

$$r_{\chi }=\sqrt{\frac{\ln\left(\frac{d_{\min}}{\tau }\right)}{\zeta_{\chi }^{\min}}}.$$ 29

In eqs 28 and 29, τ is the desired accuracy for the integral calculation and *d*_min_ is the contraction coefficient of the primitive basis function with the smallest exponent, ζ_χ_^min^, contributing to the AO χ. For a given atom, the largest *r*_χ_, denoted as *r*_*a*_, defines its atomic radius. Both, *r*_ϕ_ and *r*_*a*_, are atomic properties and define the interaction regions in the following way: If the distance $\overset{®}{AC}$ is larger than *r*_ϕ_ + *r*_*a*_ and the distance $\overset{®}{BC}$ is larger than *r*_ϕ_ + *r*_*b*_, the double asymptotic expansion for the ERI ⟨ab∥c̅⟩ will be used. In other words, both atoms, A⃗ and B⃗ , are outside the near-field region of the electrostatic potential $\phi_{\mathrm{c̅}}$ . This is schematically illustrated in Figure 1. The blue spheres in this figure represent the finite atomic basis set extensions around center A⃗ and B⃗ calculated according to eq 29. The violet sphere around center C⃗ is the near-field potential of this center according to eq 28. If the near-field potential of center C⃗ overlaps with one (or both) atomic basis set extension, as in the plot on the left of Figure 1, the corresponding ERI is a near-field ERI and must be calculated by recurrence relations^5,6^ that include the evaluation of the Boys function. On the other hand, if the violet potential sphere of center C⃗ does not overlap with the blue atomic basis set extensions, as in the right plot of Figure 1, the ⟨ab∥c̅⟩ ERI is a far-field ERI and can be calculated with the double asymptotic expansion according to eqs 24 and 25 avoiding the Boys function evaluation. This separation of ERIs into near-field and far-field ERIs holds for their derivatives, too.

**Figure 1:**
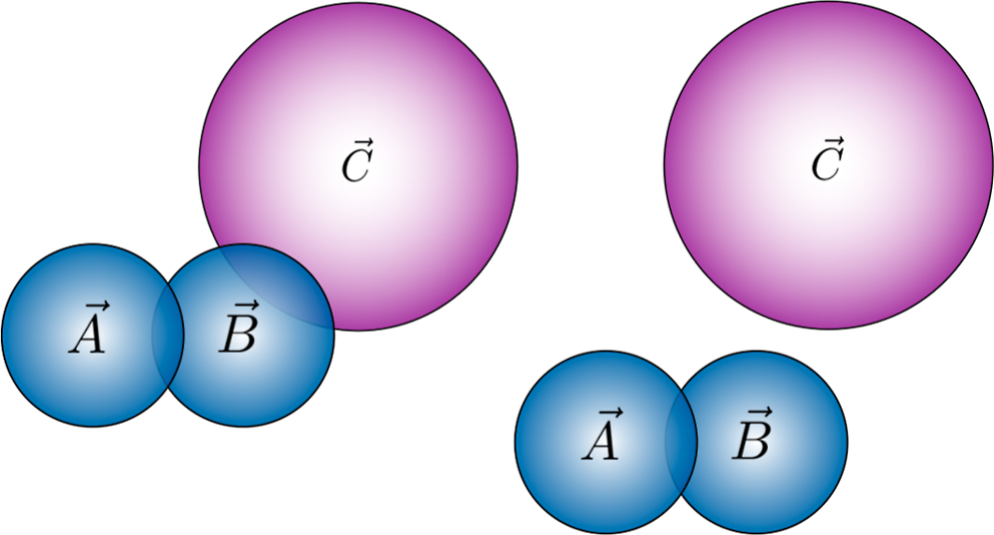
Schematic representation of the near-field (left) and far-field region (right) for ERIs. If none of the largest orbital extensions of atoms A⃗ and B⃗ overlap with the near-field region of $\phi_{\mathrm{c̅}}$ , as in the right plot, the asymptotic expansion can be applied.

### 2.3 Double Asymptotic Expansion of Three-Center ERI Second Derivatives

We start our derivation with the differential relation of Cartesian Gaussian functions according to^36^

$$\frac{\partial a}{\partial A_{i}}=2{\left({a+1}_{i}\right)}_{\left(1a\right)}-N_{i}\left(a\right)\left({a-1}_{i}\right).$$ 30

In eq 30, **a** + **1**_**i**_ indicates that the angular momentum index of **a** is augmented by 1 in its *i*-th component. The subscript indicates exponent scaling (vide infra). Analogously, **a–1**_**i**_ stipulates that the angular momentum index of **a** is decreased by 1 in its *i*-th entry. *N*_*i*_ denotes the *i*-th component of the angular momentum index, e.g. *N*_*x*_(**a**) = *a*_*x*_. The direct application of eq 30 gives the second derivatives of the near-field ERIs

31 $$\begin{array}{l} \frac{\partial^{2}\left\langle ab∥\mathrm{c̅}\right\rangle }{\partial A_{j}\partial A_{i}}=4{\left\langle \left(a+1_{i}+1_{j}\right)b∥\mathrm{c̅}\right\rangle }_{\left(2a\right)}-2N_{j}\left({a+1}_{i}\right){\left\langle \left(a+1_{i}-1_{j}\right)b∥\mathrm{c̅}\right\rangle }_{\left(1a\right)}-\\ 2N_{i}\left(a\right){\left\langle \left(a-1_{i}+1_{j}\right)b∥\mathrm{c̅}\right\rangle }_{\left(1a\right)}+N_{i}\left(a\right)N_{j}\left({a-1}_{i}\right)\left\langle \left(a-1_{i}-1_{j}\right)b∥\mathrm{c̅}\right\rangle . \end{array}$$

For the case of the mixed second derivatives, we find

32 $$\begin{array}{l} \frac{\partial^{2}\left\langle ab∥\mathrm{c̅}\right\rangle }{\partial B_{j}\partial A_{i}}=4{\left\langle \left(a+1_{i}\right)\left(b+1_{j}\right)∥\mathrm{c̅}\right\rangle }_{\left(1a+1b\right)}-2N_{j}\left(b\right){\left\langle \left(a+1_{i}\right)\left(b-1_{j}\right)∥\mathrm{c̅}\right\rangle }_{\left(1a\right)}-\\ 2N_{i}\left(a\right){\left\langle \left(a-1_{i}\right)\left(b+1_{j}\right)∥\mathrm{c̅}\right\rangle }_{\left(1b\right)}+N_{i}\left(a\right)N_{j}\left(b\right)\left\langle \left(a-1_{i}\right)\left(b-1_{j}\right)∥\mathrm{c̅}\right\rangle . \end{array}$$

In eqs 31 and 32 we introduced the following notation for the orbital exponent scaling in the ERI contraction

$${\left\langle ab∥\mathrm{c̅}\right\rangle }_{\left(2a\right)}=\overset{K_{a}}{\underset{k}{\sum }}\overset{K_{b}}{\underset{l}{\sum }}d_{k}d_{l}\zeta_{k}^{2}\left[a_{k}b_{l}∥\mathrm{c̅}\right].$$ 33

34 $${\left\langle ab∥\mathrm{c̅}\right\rangle }_{\left(1a\right)}=\sum_{k}^{K_{a}}\sum_{l}^{K_{b}}d_{k}d_{l}\zeta_{k}\left[a_{k}b_{l}∥\mathrm{c̅}\right]$$

35 $${\left\langle ab∥\mathrm{c̅}\right\rangle }_{\left(1a+1b\right)}=\sum_{k}^{K_{a}}\sum_{l}^{K_{b}}d_{k}d_{l}\zeta_{k}\zeta_{l}\left[a_{k}b_{l}∥\mathrm{c̅}\right].$$

The corresponding second derivatives with respect to center B⃗ are analogous to the ones shown in the last equations. Notice that, in this case, the orbital exponent scaling must be with ζ_*l*_ instead of ζ_*k*_, and the orbital shifting is done over **b**. These formulas can be easily extended for integral sums over the auxiliary function centers. Due to the translational invariance of the three-center ERI derivatives, only derivatives with respect to the coordinates of center A⃗ and B⃗ are needed as shown in Appendix A.

In the case of the far-field ERIs, their first derivatives are given by^37^

$$\frac{\partial \left\langle ab∥\mathrm{c̅}\right\rangle }{\partial A_{i}}∼\frac{\partial {\left\langle ab∥\mathrm{c̅}\right\rangle }^{A}}{\partial A_{i}}+\frac{\partial {\left\langle ab∥\mathrm{c̅}\right\rangle }^{B}}{\partial A_{i}}.$$ 36

Taking the derivative of the double asymptotic expansion around center A⃗ yields

37 $$\begin{array}{ll} \frac{\partial {\left\langle ab∥\mathrm{c̅}\right\rangle }^{A}}{\partial A_{i}} & ={\left(\frac{\pi }{\zeta_{\mathrm{c̅}}}\right)}^{3/2}\sum_{m}M\left(m\right)\frac{\partial T_{AC}\left(m+\mathrm{c̅}\right)}{\partial A_{i}}\left\langle a+m|b\right\rangle +\\ & {\left(\frac{\pi }{\zeta_{\mathrm{c̅}}}\right)}^{3/2}\sum_{m}M\left(m\right)T_{AC}\left(m+\mathrm{c̅}\right)\frac{\partial \left\langle a+m|b\right\rangle }{\partial A_{i}}. \end{array}$$

The derivatives of *T*_*AC*_ are given as

$$\begin{array}{ll} \frac{\partial T_{AC}\left(m+\mathrm{c̅}\right)}{\partial A_{i}} & =\frac{\partial }{\partial A_{i}}\left[{\left(\frac{\partial }{\partial C_{x}}\right)}^{m_{x}+{\mathrm{c̅}}_{x}}{\left(\frac{\partial }{\partial C_{y}}\right)}^{m_{y}+{\mathrm{c̅}}_{y}}{\left(\frac{\partial }{\partial C_{z}}\right)}^{m_{z}+{\mathrm{c̅}}_{z}}\frac{1}{\left|\mathrm{A⃗}-\mathrm{C⃗}\right|}\right]\\ & =-\frac{\partial }{\partial C_{i}}\left[{\left(\frac{\partial }{\partial C_{x}}\right)}^{m_{x}+{\mathrm{c̅}}_{x}}{\left(\frac{\partial }{\partial C_{y}}\right)}^{m_{y}+{\mathrm{c̅}}_{y}}{\left(\frac{\partial }{\partial C_{z}}\right)}^{m_{z}+{\mathrm{c̅}}_{z}}\frac{1}{\left|\mathrm{A⃗}-\mathrm{C⃗}\right|}\right]\\ & =-T_{AC}\left(m+\mathrm{c̅}+{1̅}_{i}\right). \end{array}$$ 38

For the overlap-like integral derivative in eq 37 follows directly from eq 30

39 $$\begin{array}{ll} \frac{\partial \langle a+m|b\rangle }{\partial A_{i}} & =2\langle a+1_{i}+m|{b\rangle }_{\left(1a\right)}-N_{i}\left(a+m\right)\langle a-1_{i}+m|b\rangle \\ & =2\langle a+1_{i}+m|{b\rangle }_{\left(1a\right)}-N_{i}\left(a\right)\langle a-1_{i}+m|b\rangle -m_{i}\langle a-1_{i}+m|b\rangle . \end{array}$$

The here used orbital exponent scaling is analog to the ones introduced in eqs 33–35. It is given by

40 $${\left\langle a+m|b\right\rangle }_{\left(1a\right)}=\sum_{k}^{K_{a}}\sum_{l}^{K_{b}}d_{k}d_{l}\zeta_{k}\left[a_{k}+m|b_{l}\right]$$

Substituting eqs 38 and 39 into eq 37 yields

41 $$\begin{array}{ll} \frac{\partial {\left\langle ab∥\mathrm{c̅}\right\rangle }^{A}}{\partial A_{i}} & =-{\left(\frac{\pi }{\zeta_{\mathrm{c̅}}}\right)}^{3/2}\sum_{m}M\left(m\right)T_{AC}\left(m+\mathrm{c̅}+{1̅}_{i}\right)\left\langle a+m|b\right\rangle +\\ & 2{\left(\frac{\pi }{\zeta_{\mathrm{c̅}}}\right)}^{3/2}\sum_{m}M\left(m\right)T_{AC}\left(m+\mathrm{c̅}\right){\left\langle a+1_{i}+m|b\right\rangle }_{\left(1a\right)}-\\ & N_{i}\left(a\right){\left(\frac{\pi }{\zeta_{\mathrm{c̅}}}\right)}^{3/2}\sum_{m}M\left(m\right)T_{AC}\left(m+\mathrm{c̅}\right)\left\langle a-1_{i}+m|b\right\rangle -\\ & {\left(\frac{\pi }{\zeta_{\mathrm{c̅}}}\right)}^{3/2}\sum_{m}M\left(m\right)m_{i}T_{AC}\left(m+\mathrm{c̅}\right),\left\langle a-1_{i}+m|b\right\rangle . \end{array}$$

Because the last term in eq 41 vanishes for *m*_*i*_ = 0 we can start this sum from *m*_*i*_ = 1. Substituting this summation index to start from **m** = **0** again reveals that the first and last term in eq 41 cancel each other out. Thus, we find as final expression for the first derivatives of the double asymptotic expanded three-center ERIs

42 $$\begin{array}{ll} \frac{\partial {\left\langle ab∥\mathrm{c̅}\right\rangle }^{A}}{\partial A_{i}} & =2{\left(\frac{\pi }{\zeta_{\mathrm{c̅}}}\right)}^{3/2}\sum_{m}M\left(m\right)T_{AC}\left(m+\mathrm{c̅}\right){\left\langle a+1_{i}+m|b\right\rangle }_{\left(1a\right)}-\\ & N_{i}\left(a\right){\left(\frac{\pi }{\zeta_{\mathrm{c̅}}}\right)}^{3/2}\sum_{m}M\left(m\right)T_{AC}\left(m+\mathrm{c̅}\right)\left\langle a-1_{i}+m|b\right\rangle . \end{array}$$

Note that this result is the same as if the derivatives were first calculated and then asymptotically expanded. Therefore, differentiation and double asymptotic expansion are commutative, as expected.

The corresponding first derivatives for the double asymptotic ERI expansion around center B⃗ can be derived in a similar way. It is given by

43 $$\begin{array}{ll} \frac{\partial {\left\langle ab∥\mathrm{c̅}\right\rangle }^{B}}{\partial A_{i}} & =2{\left(\frac{\pi }{\zeta_{\mathrm{c̅}}}\right)}^{3/2}\sum_{m}M\left(m\right)T_{BC}\left(m+\mathrm{c̅}\right){\left\langle {a+1}_{i}|b+m\right\rangle }_{\left(1a\right)}-\\ & N_{i}\left(a\right){\left(\frac{\pi }{\zeta_{\mathrm{c̅}}}\right)}^{3/2}\sum_{m}M\left(m\right)T_{BC}\left(m+\mathrm{c̅}\right)\left\langle {a-1}_{i}|b+m\right\rangle . \end{array}$$

The derivatives with respect to center B⃗ are very similar to the ones with respect to center A⃗ . They are given by the following expressions

44 $$\begin{array}{ll} \frac{\partial {\left\langle ab∥\mathrm{c̅}\right\rangle }^{A}}{\partial B_{i}} & =2{\left(\frac{\pi }{\zeta_{\mathrm{c̅}}}\right)}^{3/2}\sum_{m}M\left(m\right)T_{AC}\left(m+\mathrm{c̅}\right){\left\langle a+m|{b+1}_{i}\right\rangle }_{\left(1b\right)}-\\ & N_{i}\left(b\right){\left(\frac{\pi }{\zeta_{\mathrm{c̅}}}\right)}^{3/2}\sum_{m}M\left(m\right)T_{AC}\left(m+\mathrm{c̅}\right),\left\langle a+m|{b-1}_{i}\right\rangle \end{array}$$

45 $$\begin{array}{ll} \frac{\partial {\left\langle ab∥\mathrm{c̅}\right\rangle }^{B}}{\partial B_{i}} & =2{\left(\frac{\pi }{\zeta_{\mathrm{c̅}}}\right)}^{3/2}\sum_{m}M\left(m\right)T_{BC}\left(m+\mathrm{c̅}\right){\left\langle a|b+1_{i}+m\right\rangle }_{\left(1b\right)}-\\ & N_{i}\left(b\right){\left(\frac{\pi }{\zeta_{\mathrm{c̅}}}\right)}^{3/2}\sum_{m}M\left(m\right)T_{BC}\left(m+\mathrm{c̅}\right),\left\langle a|b-1_{i}+m\right\rangle . \end{array}$$

Given that the differentiation and the double asymptotic expansion commute, it is straightforward to obtain the expressions for the second derivatives of the expanded ERIs. Again only derivatives with respect to the coordinates of center A⃗ and B⃗ are needed due to the translational invariance. The explicit formulas for the second ERI derivatives with respect to center C⃗ are given in Appendix A.

The double asymptotic expansion of the second ERI derivatives with respect to center A⃗ are calculated as

46 $$\frac{\partial^{2}\left\langle ab∥\mathrm{c̅}\right\rangle }{\partial A_{j}\partial A_{i}}∼\frac{\partial^{2}{\left\langle ab∥\mathrm{c̅}\right\rangle }^{A}}{\partial A_{j}\partial A_{i}}+\frac{\partial^{2}{\left\langle ab∥\mathrm{c̅}\right\rangle }^{B}}{\partial A_{j}\partial A_{i}}$$

For the case of the second derivatives of the double asymptotic expansion centered on A⃗ we obtain

47 $$\begin{array}{ll} \frac{\partial^{2}{\left\langle ab∥\mathrm{c̅}\right\rangle }^{A}}{\partial A_{j}\partial A_{i}} & =4{\left(\frac{\pi }{\zeta_{\mathrm{c̅}}}\right)}^{3/2}\sum_{m}M\left(m\right)T_{AC}\left(m+\mathrm{c̅}\right){\left\langle a+1_{i}+1_{j}+m|b\right\rangle }_{\left(2a\right)}-\\ & 2N_{j}\left({a+1}_{i}\right){\left(\frac{\pi }{\zeta_{\mathrm{c̅}}}\right)}^{3/2}\sum_{m}M\left(m\right)T_{AC}\left(m+\mathrm{c̅}\right){\left\langle a+1_{i}-1_{j}+m|b\right\rangle }_{\left(1a\right)}-\\ & 2N_{i}\left(a\right){\left(\frac{\pi }{\zeta_{\mathrm{c̅}}}\right)}^{3/2}\sum_{m}M\left(m\right)T_{AC}\left(m+\mathrm{c̅}\right){\left\langle a-1_{i}+1_{j}+m|b\right\rangle }_{\left(1a\right)}+\\ & N_{i}\left(a\right)N_{j}\left({a-1}_{i}\right){\left(\frac{\pi }{\zeta_{\mathrm{c̅}}}\right)}^{3/2}\sum_{m}M\left(m\right)T_{AC}\left(m+\mathrm{c̅}\right)\left\langle a-1_{i}-1_{j}+m|b\right\rangle . \end{array}$$

Similar, the second derivatives of the double asymptotic expansion centered on B⃗ are given by

48 $$\begin{array}{ll} \frac{\partial^{2}{\left\langle ab∥\mathrm{c̅}\right\rangle }^{B}}{\partial A_{j}\partial A_{i}} & =4{\left(\frac{\pi }{\zeta_{\mathrm{c̅}}}\right)}^{3/2}\sum_{m}M\left(m\right)T_{BC}\left(m+\mathrm{c̅}\right){\left\langle a+1_{i}+1_{j}|b+m\right\rangle }_{\left(2a\right)}-\\ & 2N_{j}\left({a+1}_{i}\right){\left(\frac{\pi }{\zeta_{\mathrm{c̅}}}\right)}^{3/2}\sum_{m}M\left(m\right)T_{BC}\left(m+\mathrm{c̅}\right){\left\langle a+1_{i}-1_{j}|b+m\right\rangle }_{\left(1a\right)}-\\ & 2N_{i}\left(a\right){\left(\frac{\pi }{\zeta_{\mathrm{c̅}}}\right)}^{3/2}\sum_{m}M\left(m\right)T_{BC}\left(m+\mathrm{c̅}\right){\left\langle a-1_{i}+1_{j}|b+m\right\rangle }_{\left(1a\right)}+\\ & N_{i}\left(a\right)N_{j}\left({a-1}_{i}\right){\left(\frac{\pi }{\zeta_{\mathrm{c̅}}}\right)}^{3/2}\sum_{m}M\left(m\right)T_{BC}\left(m+\mathrm{c̅}\right)\left\langle a-1_{i}-1_{j}|b+m\right\rangle . \end{array}$$

The corresponding working equations for the double asymptotically expanded three-center ERI second derivatives with respect to center B⃗ are

49 $$\begin{array}{ll} \frac{\partial^{2}{\left\langle ab∥\mathrm{c̅}\right\rangle }^{A}}{\partial B_{j}\partial B_{i}} & =4{\left(\frac{\pi }{\zeta_{\mathrm{c̅}}}\right)}^{3/2}\sum_{m}M\left(m\right)T_{AC}\left(m+\mathrm{c̅}\right){\left\langle a+m|b+1_{i}+1_{j}\right\rangle }_{\left(2b\right)}-\\ & 2N_{j}\left({b+1}_{i}\right){\left(\frac{\pi }{\zeta_{\mathrm{c̅}}}\right)}^{3/2}\sum_{m}M\left(m\right)T_{AC}\left(m+\mathrm{c̅}\right){\left\langle a+m|b+1_{i}-1_{j}\right\rangle }_{\left(1b\right)}-\\ & 2N_{i}\left(b\right){\left(\frac{\pi }{\zeta_{\mathrm{c̅}}}\right)}^{3/2}\sum_{m}M\left(m\right)T_{AC}\left(m+\mathrm{c̅}\right){\left\langle a+m|b-1_{i}+1_{j}\right\rangle }_{\left(1b\right)}+\\ & N_{i}\left(b\right)N_{j}\left({b-1}_{i}\right){\left(\frac{\pi }{\zeta_{\mathrm{c̅}}}\right)}^{3/2}\sum_{m}M\left(m\right)T_{AC}\left(m+\mathrm{c̅}\right)\left\langle a+m|b-1_{i}-1_{j}\right\rangle , \end{array}$$

and

50 $$\begin{array}{ll} \frac{\partial^{2}{\left\langle ab∥\mathrm{c̅}\right\rangle }^{B}}{\partial B_{j}\partial B_{i}} & =4{\left(\frac{\pi }{\zeta_{\mathrm{c̅}}}\right)}^{3/2}\sum_{m}M\left(m\right)T_{BC}\left(m+\mathrm{c̅}\right){\left\langle a|b+1_{i}+1_{j}+m\right\rangle }_{\left(2b\right)}-\\ & 2N_{j}\left({b+1}_{i}\right){\left(\frac{\pi }{\zeta_{\mathrm{c̅}}}\right)}^{3/2}\sum_{m}M\left(m\right)T_{BC}\left(m+\mathrm{c̅}\right){\left\langle a|b+1_{i}-1_{j}+m\right\rangle }_{\left(1b\right)}-\\ & 2N_{i}\left(b\right){\left(\frac{\pi }{\zeta_{\mathrm{c̅}}}\right)}^{3/2}\sum_{m}M\left(m\right)T_{BC}\left(m+\mathrm{c̅}\right){\left\langle a|b-1_{i}+1_{j}+m\right\rangle }_{\left(1b\right)}+\\ & N_{i}\left(b\right)N_{j}\left({b-1}_{i}\right){\left(\frac{\pi }{\zeta_{\mathrm{c̅}}}\right)}^{3/2}\sum_{m}M\left(m\right)T_{BC}\left(m+\mathrm{c̅}\right)\left\langle a|b-1_{i}-1_{j}+m\right\rangle . \end{array}$$

For the case of the mixed second derivatives of the double asymptotically expanded ERIs centered on A⃗ and B⃗ , respectively, we obtain the following working expressions

51 $$\begin{array}{ll} \frac{\partial^{2}{\left\langle ab∥\mathrm{c̅}\right\rangle }^{A}}{\partial B_{j}\partial A_{i}} & =4{\left(\frac{\pi }{\zeta_{\mathrm{c̅}}}\right)}^{3/2}\sum_{m}M\left(m\right)T_{AC}\left(m+\mathrm{c̅}\right){\left\langle a+1_{i}+m|{b+1}_{j}\right\rangle }_{\left(1a+1b\right)}-\\ & 2N_{j}\left(b\right){\left(\frac{\pi }{\zeta_{\mathrm{c̅}}}\right)}^{3/2}\sum_{m}M\left(m\right)T_{AC}\left(m+\mathrm{c̅}\right){\left\langle a+1_{i}+m|{b-1}_{j}\right\rangle }_{\left(1a\right)}-\\ & 2N_{i}\left(a\right){\left(\frac{\pi }{\zeta_{\mathrm{c̅}}}\right)}^{3/2}\sum_{m}M\left(m\right)T_{AC}\left(m+\mathrm{c̅}\right){\left\langle a-1_{i}+m|{b+1}_{j}\right\rangle }_{\left(1b\right)}+\\ & N_{i}\left(a\right)N_{j}\left(b\right){\left(\frac{\pi }{\zeta_{\mathrm{c̅}}}\right)}^{3/2}\sum_{m}M\left(m\right)T_{AC}\left(m+\mathrm{c̅}\right)\left\langle a-1_{i}+m|{b-1}_{j}\right\rangle , \end{array}$$

and

52 $$\begin{array}{ll} \frac{\partial^{2}{\left\langle ab∥\mathrm{c̅}\right\rangle }^{B}}{\partial B_{j}\partial A_{i}} & =4{\left(\frac{\pi }{\zeta_{\mathrm{c̅}}}\right)}^{3/2}\sum_{m}M\left(m\right)T_{BC}\left(m+\mathrm{c̅}\right){\left\langle {a+1}_{i}|b+1_{j}+m\right\rangle }_{\left(1a+1b\right)}-\\ & 2N_{j}\left(b\right){\left(\frac{\pi }{\zeta_{\mathrm{c̅}}}\right)}^{3/2}\sum_{m}M\left(m\right)T_{BC}\left(m+\mathrm{c̅}\right){\left\langle {a+1}_{i}|b-1_{j}+m\right\rangle }_{\left(1a\right)}-\\ & 2N_{i}\left(a\right){\left(\frac{\pi }{\zeta_{\mathrm{c̅}}}\right)}^{3/2}\sum_{m}M\left(m\right)T_{BC}\left(m+\mathrm{c̅}\right){\left\langle {a-1}_{i}|b+1_{j}+m\right\rangle }_{\left(1b\right)}+\\ & N_{i}\left(a\right)N_{j}\left(b\right){\left(\frac{\pi }{\zeta_{\mathrm{c̅}}}\right)}^{3/2}\sum_{m}M\left(m\right)T_{BC}\left(m+\mathrm{c̅}\right)\left\langle {a-1}_{i}|b-1_{j}+m\right\rangle . \end{array}$$

For the orbital exponent scaled overlap-like integrals, the following notations are used

$$\langle a+m|{b\rangle }_{\left(2a\right)}=\overset{K_{a}}{\underset{k}{\sum }}\overset{K_{b}}{\underset{l}{\sum }}d_{k}d_{l}\zeta_{k}^{2}\left[a_{k}+m|b_{l}\right],$$ 53

$$\langle a+m|{b\rangle }_{\left(1a+1b\right)}=\overset{K_{a}}{\underset{k}{\sum }}\overset{K_{b}}{\underset{l}{\sum }}d_{k}d_{l}\zeta_{k}\zeta_{l}\left[a_{k}+m|b_{l}\right].$$ 54

These working equations for the double asymptotically expanded second derivatives of three-center ERIs are symmetric with respect to the interchange of index *i* with *j*. We point out once again that these expressions are free of the Boys function and are low-order scaling due to the two-center overlap-like integrals. The here presented working equations for the double asymptotic expansion of the three-center ERI second derivatives have been implemented into the deMon2k code (6.3 version). In the following sections, we explore how the double asymptotic expansion of the ERI second derivatives improves the computational timing in the calculation of the skeleton Hessian matrix (eq 17).

## 3 Computational Details

To benchmark the double asymptotic expansion of the three-center ERI second derivatives, we calculated the skeleton Hessian matrix for a variety of systems with up to 43,000 basis functions. These benchmark systems consist of (a) linear alkane chains with 50, 100, 150, 200, 250, 300, 400, 600, 800, 1000, 1200, 1400, and 1600 carbon atoms; (b) DNA fragments with 1, 2, 4, 8, and 16 adenine-thymine base pairs;^38^ (c) fullerene molecules with 180, 240, 540, 720, and 960 carbon atoms; and (d) hydrogen saturated mobil-5 type (ZSM-5) zeolite fragments with sizes ranging from 376 to more than 1400 atoms.^39^ Characteristic structures of these systems are presented in Figure 2. All calculations were performed with the LCGTO-ADFT program deMon2k version 6.3 code.^21,40–42^ We employed the PBE^43^ exchange–correlation functional in combination with an all-electron double-ζ valence-polarization (DZVP) orbital basis set^44^ and the automatically generated GEN-A2* auxiliary basis set.^45^ The SCF convergence criterion was set to 10^–5^ a.u. for the MinMax energy bounds^46^ and 10^–4^ a.u. for the charge density fitting. The minimal residue (MINRES) method^39^ was used for the solution of the fitting equation systems. The ERI accuracy threshold τ in eqs 28 and 29 was set to its default value of 10^–10^ atomic units. Using the default asymptotic expansion order of 8, energy derivative accuracies of 10^–6^ a.u. or better are achieved. This means that individual elements of the skeleton Hessian matrix calculated with the double asymptotic expansion agree to 10^–6^ or better with their reference counterparts without this expansion. This translates to frequency agreements between DIRECT and ASYMPTOTIC calculation better than 0.1 cm^–1^.To keep the computational time reasonable, we performed the benchmark calculations in two steps. In the first step only single point SCF calculations were performed. To this end, we used the high-performance computing (HPC) facility NARVAL of the Digital Research Alliance of Canada. The standard compute nodes of NARVAL are 2 × 32 cores AMD EPYC 7532 CPUs with 4 GB random access memory (RAM) per core. To overcome ADFT RAM limitations in parallel runs of big systems we use an algorithmic scheme based on shared memory matrices. In this scheme all cores of one compute node share the same physical field for a matrix. This is different to the more regular scheme of message passing interface (MPI) where each core has its own copy of a matrix. Working with shared memory matrices requires extra programming to handle cases in which different cores modify the same part of a matrix. In a second step, the skeleton Hessian matrix was calculated from the deMon2k restart file on the large memory node of our local cluster at Cinvestav. This node has 2 × 12 core Intel Xeon Gold 6226 CPUs with together 768 GB RAM. All reported timings refer to this architecture.

## 4 Results and Discussion

In this section, we present parallel (24 cores) timings for the skeleton Hessian matrix calculations. For all molecules of the test set, the second derivatives of the ERIs were obtained using two different approaches available in deMon2k. The first one, named DIRECT, is the approach in which all ERIs are calculated on the fly by integral recurrence relations. The second approach, called ASYMPTOTIC, uses the DIRECT approach to calculate the second derivatives for the near-field ERIs, while the second ERI derivatives in the far-field region are calculated using the double asymptotic expansion as described in Section 2. For alkane chains a third option was used, too. This is the CONVENTIONAL approach, in which ERIs and their derivatives are calculated once and stored in core or hard disk. Obviously, this approach is memory limited and, therefore, only applicable to relative small systems. As a result, the CONVENTIONAL approach can only be applied to alkane chains with up to 300 carbon atoms. On the other hand, the DIRECT and ASYMPTOTIC approaches were used for the calculation of skeleton Hessian matrices for alkane chains up to 1600 carbon atoms. Due to this different size ranges we also used different intervals, namely 50 carbon atoms for the CONVENTIONAL and 200 carbon atoms for the DIRECT and ASYMPTOTIC approaches. The timings obtained for the skeleton Hessian matrix calculations of the alkane chain molecules with respect to the number of basis functions are graphically depicted in Figure 3. In this figure, the timings obtained for the CONVENTIONAL approach are given by black ticks, whereas the timings for the DIRECT and ASYMPTOTIC approaches are given by blue crosses and red triangles, respectively. To guide the eye we connected the data points of the depicted approaches in Figure 3. As this figure shows, the ASYMPTOTIC approach clearly outperforms the DIRECT one. For the largest system, the computer time is roughly halved by the ASYMPTOTIC approach. Even for relatively small systems, e.g. the alkane with 200 carbon atoms (5412 basis functions) the ASYMPTOTIC approach is with 376 s faster than the CONVENTIONAL (598 s) and the DIRECT (714 s) one. At first glance surprisingly, the scaling is, for all 3 second derivative ERI approaches, nearly quadratic. Closer inspection reveals that this scaling arises from the (quadratic) number of skeleton Hessian matrix elements that need to be calculated and not from the (subquadratic) computational scaling for each of these elements. We attribute this to the integral screening which is particularly efficient for linear type systems. As a result, the three-center ERI second derivative calculation in alkane chains is computationally not dominant for the skeleton Hessian matrix calculation.

**Figure 2:**
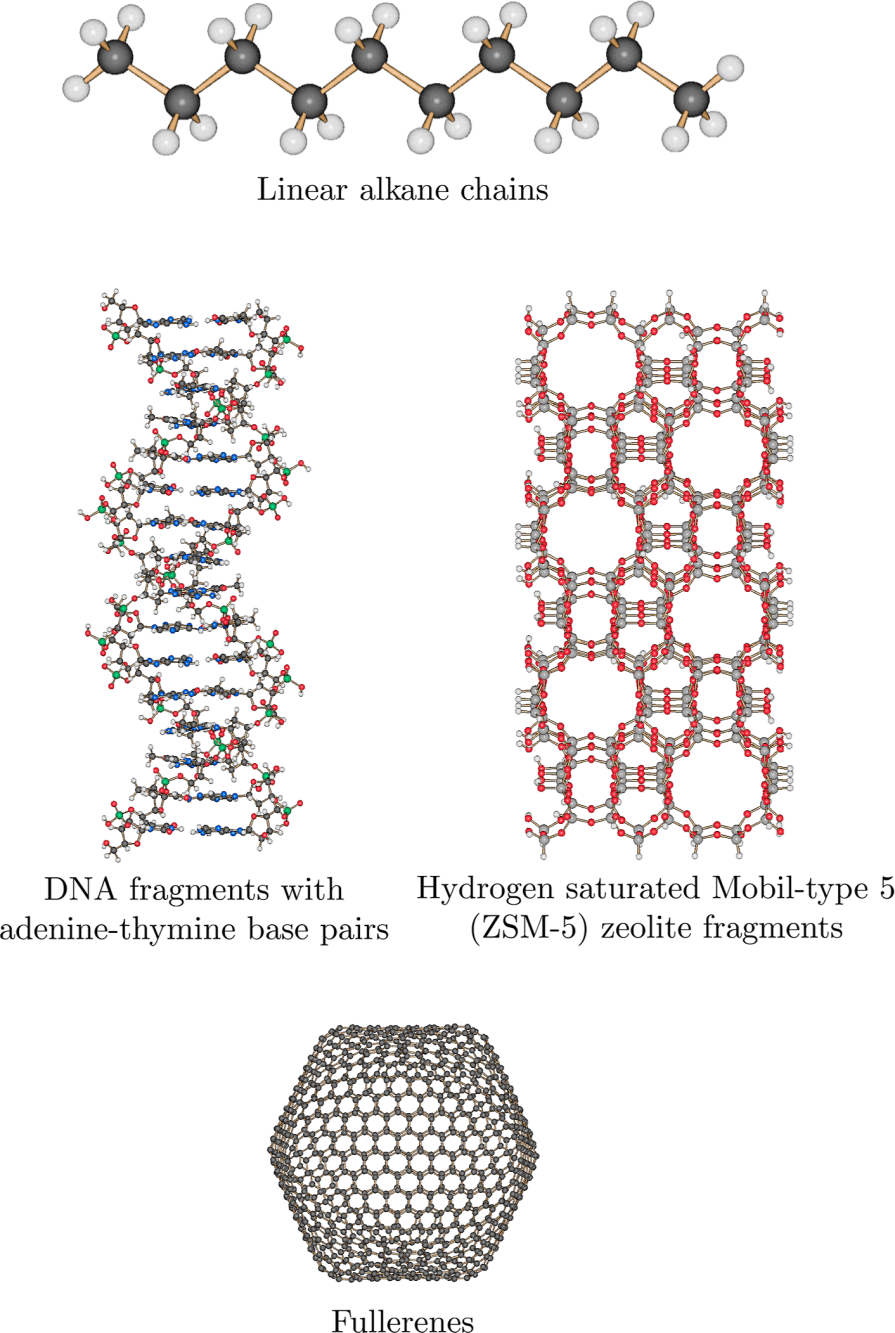
Characteristic structures of benchmark systems. Top: Linear alkane chains C_*n*_H_2(*n*+1)_, with *n* = 50, 100, 150, 200, 250, 300, 400, 600, 800, 1000, 1200, 1400, 1600, where *n* is the number of carbon atoms; Middle left: DNA fragments with 1, 2, 4, 8, and 16 adenine-thymine base pairs; Middle right: Hydrogen saturated ZSM-5 zeolite fragments; Bottom: Fullerenes C_*n*_, with *n* = 180, 240, 540, 720, 960. The used atom colors are white for hydrogen, black for carbon, red for oxygen, blue for nitrogen, gray for silicon and green for phosphorus atoms, respectively.

**Figure 3:**
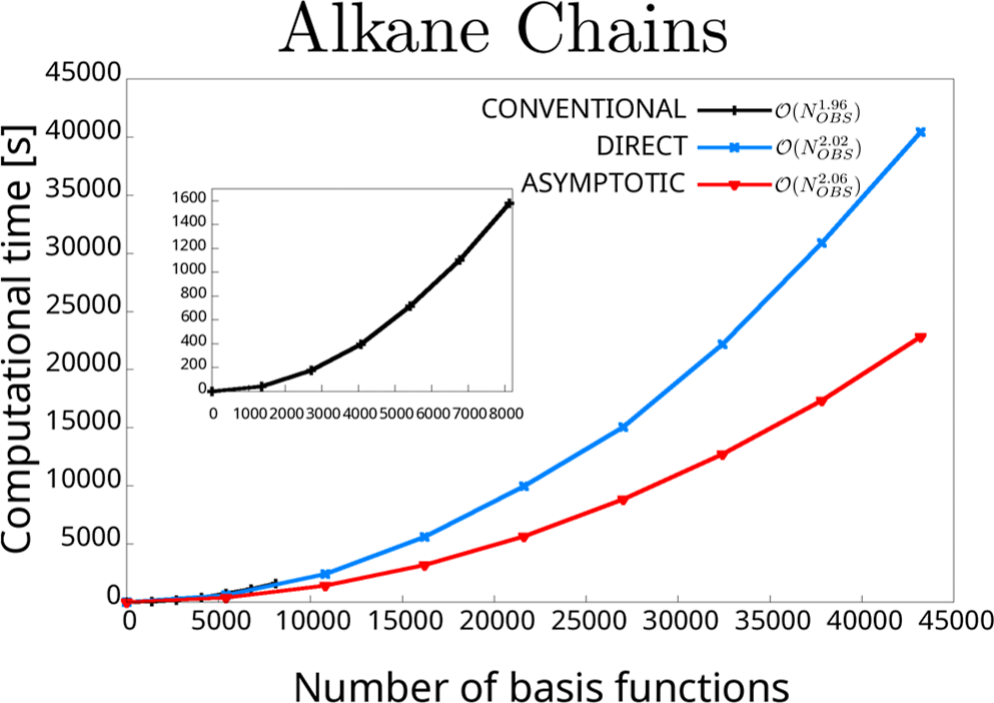
Timings (in seconds) of the skeleton Hessian matrix calculations for linear alkane chains with respect to the number of basis functions. The CONVENTIONAL option is used for linear alkane chains with 50, 100, 150, 200, 250, and 300 carbon atoms. DIRECT and ASYMPTOTIC ERI options are used for linear alkane chains with 200, 400, 600, 800, 1000, 1200, 1400, and 1600 carbon atoms. See the text for computational details.

The timings for the skeleton Hessian matrix calculations of DNA fragments with 1, 2, 4, 8, and 16 adenine-thymine base pairs (see Figure 2 for a structural template) are plotted in the top graph of Figure 4. The color code is the same as in the alkane graph, Figure 3. Although the studied DNA fragments are significantly smaller in basis set size than the alkane chains, their three-center ERI second derivative scaling shows more variation. We attribute this to the nonlinear structure of the DNA fragments. As the top graph in Figure 4 shows, a slightly larger than quadratic scaling is found for the DIRECT approach. Using the ASYMPTOTIC approach this scaling is substantially reduced to 1.59. This indicates that in the DNA fragments the three-center ERI second derivative calculation dominates the scaling of the skeleton Hessian matrix calculations. The computational time saving by the double asymptotic ERI expansion for the largest DNA fragment with 16 base pairs is about 40% and, therefore, at around 12,000 basis functions already significant for the skeleton Hessian matrix calculations.

**Figure 4:**
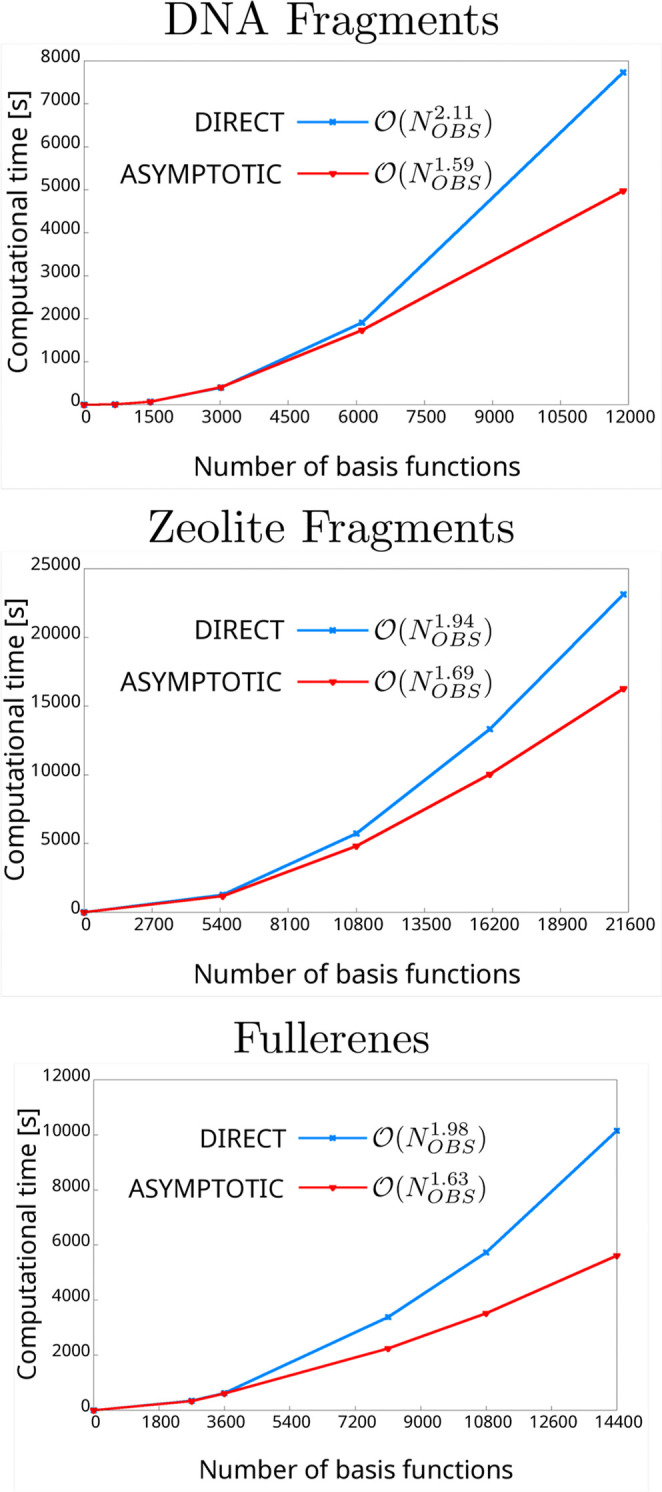
Timings (in seconds) of the skeleton Hessian matrix calculations for DNA fragments (top), hydrogen saturated ZSM-5 zeolite fragments (middle) and fullerenes (bottom). The DIRECT and ASYMPTOTIC ERI options are used. See the text for computational details.

The next class of systems we employed in our benchmark calculations are hydrogen saturated ZSM-5 zeolite fragments (see Figure 2 for structure template) with sizes ranging from 376 to more than 1400 atoms. The obtained timings for the skeleton Hessian matrix calculations are plotted in the middle graph of Figure 4. In this case the smallest system consists of about 5400 basis functions and the largest one of almost 21,500 basis functions. The obtained scaling is similar to the one of the DNA fragments, namely nearly quadratic for the DIRECT approach (1.94) and subquadratic for the ASYMPTOTIC one. Note, however, that the ASYMPTOTIC scaling of 1.69 for the zeolite fragments is larger than that for the DNA fragments (1.59). We attribute this to the denser three-dimensional atom packing in the zeolites. This is also the reason for the only moderate computational time saving (∼30%) for the largest ZSM-5 system with the ASYMPTOTIC approach.

The last system type we benchmarked for three-center ERI second derivative calculations are fullerenes with 180, 240, 540, 720, and 960 carbon atoms (see Figure 2 for structure template). The computational timings for the fullerenes skeleton Hessian matrix calculations are depicted in the bottom of Figure 4. Again, we find a significant scaling reduction when using the double asymptotic expansion for the ERI second derivatives. This reduction is somehow in between the one found for the DNA fragments and the ZSM-5 fragments. Characteristic for fullerenes is the strong growth of the difference between the DIRECT and the ASYMPTOTIC approaches. Closer inspection reveals that this is structural related. Whereas the ERI second derivative calculations in smaller fullerenes is similar to three-dimensional structures, it turns, with increasing system diameter, into a more two-dimensional behavior. As a result, the computational time saving with the ASYMPTOTIC approach for the skeleton Hessian matrix calculation is already for C_960_ more than 50%.

## 5 Conclusions

The working equations for the double asymptotic expansion of three-center ERI second derivatives have been derived. They are implemented in deMon2k and benchmarked by ADFT skeleton Hessian matrix calculations. The benchmark results show that the double asymptotic expansion of three-center ERI second derivatives can reduce significantly, up to a factor of 2 for some of the here studied systems, the computational time for the skeleton Hessian matrix calculations. The savings with the ASYMPTOTIC approach usually start at around 5000 basis functions and increase continuously with system size. Surprisingly, these savings do not always influence the scaling. For alkane chains a quadratic scaling independent of the used approach is observed. We attribute this to the linear structure of these chains that make integral screening through basis function products particularly efficient. In combination with the shared exponent primitive Hermite Gaussian ABS structure an intrinsically subquadratic scaling for three-center ERI and ERI derivative calculations is achieved. Thus, the observed quadratic scaling for alkane chains reflects the quadratic nature of the skeleton Hessian matrix. This situation changes for the studied nonlinear systems like the DNA and ZSM-5 fragments as well as the fullerenes. Here the double asymptotic expansion of the three-center ERI second derivatives reduces the scaling of the skeleton Hessian matrix calculations. This underlines the dominance of this computational step for the skeleton Hessian matrix calculations in these systems. The observed subquadratic scaling for the ASYMPTOTIC approach opens the possibility of Hessian matrix calculations for systems with 50,000 basis functions and beyond. Such an implementation is currently under development in our laboratories.

## Figures and Tables

**A1 figA1:**
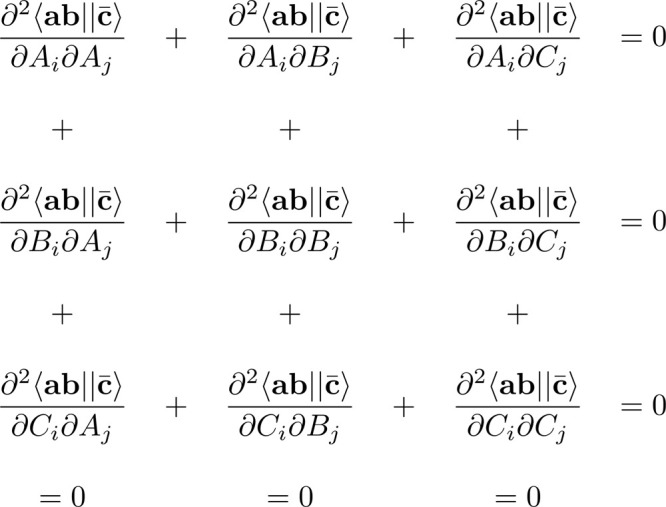
Translational invariances for second derivatives of three-center electron repulsion integrals.

## Data Availability

In the manuscript is specified the used software (https://www.demon-software.com). Detailed settings for all calculations are outlined in the Computational details section, along with the software version.

## Appendix A: Translation invariance for three-center ERI 2nd derivatives

In this appendix the explicit formulas for the calculation of second ERI derivatives with respect to center C⃗ are presented. These derivatives, for both near- and far-field ERI second derivatives, are calculated by translational invariance according to the relations given in Figure [Fig figA1].

From the vertical and horizontal translational invariance conditions follows for the mixed second ERI derivatives

55
$$\frac{\partial^{2}\left\langle ab∥\mathrm{c̅}\right\rangle }{\partial A_{i}\partial C_{j}}=-\frac{\partial^{2}\left\langle ab∥\mathrm{c̅}\right\rangle }{\partial A_{i}\partial A_{j}}-\frac{\partial^{2}\left\langle ab∥\mathrm{c̅}\right\rangle }{\partial A_{i}\partial B_{j}}$$

56
$$\frac{\partial^{2}\left\langle ab∥\mathrm{c̅}\right\rangle }{\partial B_{i}\partial C_{j}}=-\frac{\partial^{2}\left\langle ab∥\mathrm{c̅}\right\rangle }{\partial B_{i}\partial A_{j}}-\frac{\partial^{2}\left\langle ab∥\mathrm{c̅}\right\rangle }{\partial B_{i}\partial B_{j}}$$

57
$$\frac{\partial^{2}\left\langle ab∥\mathrm{c̅}\right\rangle }{\partial C_{i}\partial A_{j}}=-\frac{\partial^{2}\left\langle ab∥\mathrm{c̅}\right\rangle }{\partial A_{i}\partial A_{j}}-\frac{\partial^{2}\left\langle ab∥\mathrm{c̅}\right\rangle }{\partial B_{i}\partial A_{j}}$$

58
$$\frac{\partial^{2}\left\langle ab∥\mathrm{c̅}\right\rangle }{\partial C_{i}\partial B_{j}}=-\frac{\partial^{2}\left\langle ab∥\mathrm{c̅}\right\rangle }{\partial A_{i}\partial B_{j}}-\frac{\partial^{2}\left\langle ab∥\mathrm{c̅}\right\rangle }{\partial B_{i}\partial B_{j}}$$

For the pure second ERI derivative with respect to center C⃗ follows from the last horizontal translational invariance condition

59
$$\frac{\partial^{2}\left\langle ab∥\mathrm{c̅}\right\rangle }{\partial C_{i}\partial C_{j}}=-\frac{\partial^{2}\left\langle ab∥\mathrm{c̅}\right\rangle }{\partial C_{i}\partial A_{j}}-\frac{\partial^{2}\left\langle ab∥\mathrm{c̅}\right\rangle }{\partial C_{i}\partial B_{j}}$$

60
$$=\frac{\partial^{2}\left\langle ab∥\mathrm{c̅}\right\rangle }{\partial A_{i}\partial A_{j}}+\frac{\partial^{2}\left\langle ab∥\mathrm{c̅}\right\rangle }{\partial B_{i}\partial A_{j}}+\frac{\partial^{2}\left\langle ab∥\mathrm{c̅}\right\rangle }{\partial A_{i}\partial B_{j}}+\frac{\partial^{2}\left\langle ab∥\mathrm{c̅}\right\rangle }{\partial B_{i}\partial B_{j}}$$

Therefore, as these formulas show, only the second derivatives with respect to centers A⃗ and B⃗ are needed for the calculation of three-center ERI second derivatives.
